# A Five-Year Retrospective Study: Relationship Between Cervical Spine Whiplash Injuries and Demyelinating Diseases in the Pediatric Population

**DOI:** 10.7759/cureus.104866

**Published:** 2026-03-08

**Authors:** Maya S Kardouh, Colin Van Wagoner, Jacob Coleman, Ehab Saleh

**Affiliations:** 1 Internal Medicine, Oakland University William Beaumont School of Medicine, Rochester, USA; 2 Orthopedic Surgery, Oakland University William Beaumont School of Medicine, Rochester, USA; 3 Orthopedics, Corewell Health William Beaumont University Hospital, Royal Oak, USA

**Keywords:** cervical spine whiplash injury, demyelinating disease, pediatric cervical spine, pediatric orthopedic spine, trauma pediatric

## Abstract

Objectives: Whiplash injuries are among the most common cervical spine injuries in the pediatric population. Limited literature demonstrates a potential link between cervical spine trauma and demyelinating disease, such as multiple sclerosis. These associations have primarily been reported from case-controlled studies, while cohort studies have not supported these findings. In this study, we aimed to evaluate whether a relationship exists between cervical spine trauma and the short-term development of demyelinating disease in pediatric patients. We hypothesized that patients experiencing cervical spine whiplash injuries would demonstrate subsequent demyelinating diseases.

Methods: This was a single-center retrospective chart review. Charts for patients aged 10-16 with a suspected history of cervical spine whiplash injury were electronically reviewed for any post-traumatic symptoms of demyelinating disease. Patient’s past medical history and family history were also reviewed to evaluate for any predisposing risk factors for demyelinating disease. Statistical analysis was descriptive in nature. The collected data were summarized using counts and percentages. No inferential statistical testing was performed.

Results: Of the 63 patients included for evaluation, 0 were found to have evidence of demyelinating disease following cervical spine whiplash injury. Median age was 13 years, and 52% of the patients were female.

Conclusions: The descriptive findings of this pilot study demonstrated no cases of demyelinating disease in pediatric patients experiencing cervical spine whiplash injuries within a five-year period. Further research involving larger cohorts and extended follow-up time is recommended to provide further insight regarding this potential association.

## Introduction

Whiplash injuries, which typically result from road-traffic collisions and contact sports trauma, are among the most common cervical spine injuries in the pediatric population [1-3]. While often considered benign and managed conservatively with rest, analgesics, and physical therapy, emerging evidence suggests that whiplash may have longstanding neurologic implications [4]. Previous studies have suggested a potential link between cervical spine trauma and demyelinating diseases, such as multiple sclerosis (MS). Although case-controlled studies have reported associations between head trauma and MS, cohort studies have not supported these findings, leaving the relationship unclear [5].

Recent investigations have highlighted the role of spinal cord injury in increasing MS risk, and case reports have described rapid-onset MS following whiplash, particularly in individuals with a family history of demyelinating diseases [6,7]. Trauma-induced disruption of the blood-brain barrier has been implicated in whiplash injuries, which could suggest a plausible biologic mechanism for central demyelination [8]. Additionally, nerve pathology and neuropathic pain have been documented in patients with whiplash-associated disorders, further supporting the possibility of underlying neurological sequelae [9].

Despite these observations, there is a paucity of data examining this relationship in the pediatric population. Early identification of neurological disease could significantly impact long-term outcomes [10]. The primary purpose of this study was to evaluate whether a relationship exists between cervical spine whiplash injuries and the subsequent acute development of demyelinating disease in pediatric patients. Secondary aims include determining whether affected patients had a predisposition for a demyelinating disease by analyzing their past medical history and family history. We hypothesized that pediatric patients experiencing cervical spine whiplash injuries would demonstrate the subsequent development of demyelinating disease.

## Materials and methods

This was a single-center retrospective chart review performed at Corewell Health William Beaumont University Hospital, a Level I trauma center. Institutional Review Board approval was granted for project completion. The electronic medical record was queried for patient encounters between January 2019 and January 2024. Patient inclusion criteria included the following: age 10-16 years, suspected history of cervical spine whiplash injury due to a motor vehicle or sports-related accident, and cervical spine magnetic resonance imaging (MRI) performed following the traumatic event. Exclusion criteria included patients aged younger than 10 years or older than 16 years, those with no cervical spine MRI, or those with a prior history of demyelinating disease before the suspected whiplash injury. 

Patient charts were reviewed electronically using International Classification of Diseases, Tenth Revision (ICD-10) codes to identify whiplash or whiplash-like injuries (Table 4, Appendix). These codes included those for sprain, subluxation, or dislocation of the cervical spine at initial or subsequent encounters and any associated sequelae of those events. A total of 63 patients met the inclusion criteria and matched the ICD-10 codes. Subsequently, their charts were searched with the keywords "hyperintensities," "demyelination," "numbness," and "tingling." These keywords were selected as being representative of clinical and/or radiographic findings associated with demyelinating disease.

Primary variables included age, sex, cervical spine MRI and computed tomography (CT) findings, duration between accident and onset of symptoms, demyelination symptoms experienced, family history of demyelinating disease, other physical trauma, other neurological diseases/symptoms, and treatment. Secondary variables collected included additional past medical history and family history other than demyelinating diseases.

Statistical analysis was performed using descriptive methods. Patient demographics, past medical history, family history, and clinical outcomes were summarized as counts and percentages. Given the retrospective observational design and small sample size, no hypothesis testing, group comparisons, or inferential analyses were performed. All analyses were conducted using Microsoft Excel 2024 (Microsoft, Redmond, WA), and findings are presented descriptively to provide an overview of the cohort.

## Results

A total of 63 patients were included for analysis. The median and mean patient ages at admission were 13.0 years and 13.1 years, respectively. 52% of patients were female (n = 33) (Table 1). Following the chart and imaging review using keywords, zero patients were found to have evidence of demyelinating disease. Two patients had a note of hyperintensity on cervical spine imaging, one of which was noted on cervical spine CT, which was more consistent with a subarachnoid hemorrhage rather than a demyelinating process. The other hyperintensity was identified on a cervical spine MRI, which was more consistent with an acute interspinous ligamentous sprain than a demyelinating process (Table 2). 

**Table 1:** Patient demographic data, including age and gender, are reported with mean and median values for continuous data, and raw values and percentages are provided for categorical data.

Total patients	63
Median age (Years)	13.0
Mean age (Years)	13.1
Gender	
Female	52.3% (n = 33)
Male	47.6% (n = 30)

**Table 2:** Clinical and radiographic findings regarding possible demyelinating disease.

Finding	Number of Patients (n)	Notes
Evidence of demyelinating disease	0	
Hyperintensity on cervical spine imaging	2	One on CT (subarachnoid hemorrhage); one on MRI (acute interspinous ligament sprain)

The patient cohort had a total of 87 different past medical diagnoses. The diagnoses included, but were not limited to, allergies, heart abnormalities, scoliosis, nausea, etc. Four patients had a history of depression, four had a history of headache, and three had a history of concussion. 

Regarding patients’ family history, 58.7% of patients (n = 37) did not have a documented family history of disease. For patients with a documented family history, there was a similar wide, unrelated range of diagnoses, including 66 different diagnoses. These included diabetes, heart failure, anxiety, headaches, and hyperlipidemia. The diagnoses with the highest frequency included diabetes (n = 13), asthma (n = 12), cancer (n = 11), hypertension (n = 10), and stroke (n = 6). Of note, zero patients demonstrated a family history of demyelinating disease (Table 3). 

**Table 3:** Medical and family histories are documented within patient charts and used to evaluate for possible predisposing factors related to demyelinating disease.

Past Medical History	Result	Notes
Total diagnoses	87	This included a very wide range of diagnoses, with no more than four patients having the same diagnosis.
Family History		
No documented family history	58.7% (n = 37)	
Total diagnoses	66	No evidence of family history of demyelinating disease for any patient.

## Discussion

In this five-year retrospective, single-center study of pediatric patients aged 10 to 16 years, we found no cases of pediatric cervical spine whiplash injuries that resulted in the development of demyelinating disease within a five-year period. There were insufficient findings to evaluate the time between the accident and the onset of symptoms, what symptoms were experienced, or the treatment modalities used. Represented by the broad medical and family histories, our data did not establish a clinical association between potential predisposing factors, cervical spine trauma, and the development of demyelinating disease.

The lack of clinical association is a valuable finding to compare to the current literature. As previously stated, cervical whiplash is common in children but often considered benign and managed conservatively, although recent studies state the potential for long-term effects from such injuries [1-4]. For example, Fundaun et al. demonstrated that nerve pathology and neuropathic pain are common in patients with whiplash-associated disorders, supporting the possibility of broader neurologic sequelae [9]. Similarly, Sterling emphasized the chronic disability that can follow whiplash, underscoring that these injuries cannot always be dismissed as transient [3].

A potential relationship between trauma and demyelinating disease has been explored for decades. In a systematic review and meta-analysis by Lunny et al., they found that case-control studies often suggested a link between trauma and MS, but cohort studies did not [5]. Using a population-based design, Lin et al. demonstrated that spinal cord injury was associated with increased MS risk, suggesting that more severe injuries may play a role in demyelination [6]. In contrast, large-scale studies such as those by Pfleger et al. and Goldacre et al. found no increased MS risk after head injury [11,12]. These findings parallel our own null results, supporting the interpretation that relatively mild trauma, such as pediatric whiplash, may not serve as an independent etiologic factor in MS.

Nonetheless, case reports continue to describe MS onset after whiplash, including Harris et al., who reported rapid-onset MS following a cervical acceleration-deceleration injury [7]. Poser also proposed a mechanistic framework in which trauma disrupts the blood-brain barrier, potentially initiating demyelination [8]. More recent work by Abouelmagd et al. suggested that head trauma may increase the odds of MS by approximately 30% to 40%, although heterogeneity across studies was high [13]. Similarly, Eid et al. demonstrated that adverse childhood experiences, including abuse, are associated with increased MS risk, suggesting that environmental stressors, broadly defined, may act as cofactors in disease onset [14].

Our study focused on the pediatric population, in whom early recognition of acquired demyelinating syndromes is particularly important but simultaneously less likely to present in this age group. Canavese et al. highlighted the need for close follow-up during the first year after the onset of pediatric demyelinating syndromes, as clinical course and prognosis can shift rapidly [10]. Although we found no evidence of whiplash-associated demyelination within our five-year window, longer-term surveillance is warranted to identify later-onset cases.

Taken together, the literature suggests that while trauma may play a contributory role in demyelinating disease under specific conditions, particularly in patients with genetic predisposition or concomitant environmental risk factors, it may not be a major independent cause. This question ultimately needs further investigation before receiving a clear answer. As such, the strength of this pilot study lies in suggesting an approach to understanding the relationship between whiplash injury and demyelinating disease.

Previous research shows the incidence of demyelinating disease in children 0-18 years old in the United States is 0.51 per 100,000 person-years [15]. This is in the absence of concomitant cervical spine injury. The small sample size represented by this pilot study is likely a contributor to insufficient new cases of demyelinating disease being identified. Our group recommends increased time to follow up and a greater cohort size to further investigate this relationship. 

This study has several limitations. The small sample size limited statistical power to detect modest associations, and the retrospective design presented the possibility of misclassification and missing data, such as more than half of the patients lacking documented family history. Collecting data from a single center limits generalizability. Further, data was restricted to documentation during a patient’s admission, limiting our ability to evaluate long-term sequelae of their injuries and potentially identifying later-onset demyelination. Additionally, only collecting data across five years likely further limited our sample size. Furthermore, this study relied on the charted documentation of providers in history and physicals, progress notes, and imaging reads, presenting the possibility of bias resulting from the misinterpretation of symptoms or imaging by medical personnel. Last, given this was a retrospective study, imaging was not controlled; for example, there was no control for final or follow-up MRI or CT images.

We recommend that future studies be prospective, multi-center, and adequately powered, with extended follow-up into adulthood to assess long-term outcomes. Stratification by injury severity, the incorporation of genetic and environmental risk factors, and the use of advanced imaging and biomarkers to detect subclinical demyelination would also be valuable in further clarifying any potential associations.

## Conclusions

The findings of this pilot study demonstrated that within the small cohort studied, there were no cases of demyelinating disease in pediatric patients experiencing cervical spine whiplash injuries within a five-year period. Although consistent with several large cohort studies, the complexity of demyelinating disease requires further research to clearly delineate these relationships in the pediatric population. Investigation with larger, prospective cohort studies with enhanced longitudinal follow-up will help to account for the interplay of trauma, genetic predisposition, and environmental risk factors that impact the development of demyelinating disease.

## Appendices

International classification of diseases

Table 4 shows the 10th revision of International Classification of Diseases.

**Table 4:** International classification of diseases, tenth revision (ICD-10)

ICD-10 Code	Description
S13.100A	Subluxation of unspecified cervical vertebrae, initial encounter
S13.120A	Subluxation of C1/C2 cervical vertebrae, initial encounter
S13.121A	Dislocation of C1/C2 cervical vertebrae, initial encounter
S13.121D	Dislocation of C1/C2 cervical vertebrae, subsequent encounter
S13.4XXA	Sprain of ligaments of cervical spine, initial encounter
S13.4XXD	Sprain of ligaments of cervical spine, subsequent encounter
S13.4XXS	Sprain of ligaments of cervical spine, sequela
S13.8XXA	Sprain of joints and ligaments of other parts of neck, initial encounter
S13.9XXA	Sprain of joints and ligaments of unspecified parts of neck, initial encounter
